# Small obliquely oriented cortical cerebellar infarctions are associated with cardioembolic stroke

**DOI:** 10.1186/s12883-019-1328-0

**Published:** 2019-05-18

**Authors:** Adrien Ter Schiphorst, Lavinia Tatu, Vincent Thijs, Christophe Demattei, Eric Thouvenot, Dimitri Renard

**Affiliations:** 1 Department of Neurology, Nîmes University Hospital, Hôpital Carémeau, 4, Rue du Pr Debré, 30029 Nîmes, Cedex 4 France; 2 Florey Institute of Neuroscience and Mental Health, University of Melbourne, Melbourne, Vic Australia; 3 Department of Neurology, Austin Health, Heidelberg, Vic Australia; 4 Service de Biostatistique, Epidémiologie Clinique, Santé Publique et Innovation en Méthodologie (BESPIM), Nîmes University Hospital, Nîmes, France; 5 Institut de Génomique Fonctionnelle, UMR5203, INSERM 1191, Université Montpellier, Montpellier, France

**Keywords:** Stroke, Infarction, Obliquely, Cerebellar, Cortical, Cardioembolic, Cardioembolism

## Abstract

**Background:**

A revised classification of cerebellar infarctions (CI) may uncover unrecognized associations with etiologic stroke subtypes. We hypothesized that obliquely oriented small cortical cerebellar infarction (SCCI) representing end zone infarctions on MRI would be associated with cardiac embolism.

**Methods:**

We retrospectively analyzed consecutive stroke patients recruited between January–December 2016 in our center. Analyzed baseline characteristics: sex, age, cardiovascular risk factors, history of stroke or atrial fibrillation (AF). TOAST classification was used for determining stroke subtype. Acute infarction location (anterior/posterior/mixed anterior-posterior circulation), acute uni- or multiterritorial infarction, and acute or chronic CI/SCCI/non-SCCI were assessed by MRI, and vertebrobasilar stenosis/occlusion by vessel imaging. Pre-specified analysis was also performed in patients without known high cardioembolic risk (known AF history or acute multiterritorial infarction).

**Results:**

We included 452 patients (CI in 154, isolated SCCI in 55, isolated non-SCCI in 50, and mixed SCCI/non-SCCI in 49). Both SCCI and non-SCCI were associated with AF history (SCCI, *p* = 0.021; non-SCCI, *p* = 0.004), additional acute posterior circulation infarction (*p* < 0.001 both CI-subtypes), multiterritorial infarctions (SCCI, *p* = 0.003; non-SCCI, *p* < 0.001) and cardioembolic more frequent than large-artery atherosclerosis origin (*p* < 0.001 for both CI-subtypes). SCCI was associated with older age (*p* < 0.001), whereas non-SCCI was associated with stroke history (*p* = 0.036) and vertebrobasilar stenosis/occlusion (*p* = 0.002). SCCI were older (*p* = 0.046) than non-SCCI patients, had less frequently prior stroke (*p* < 0.001), and more frequent cardioembolic infarction (*p* = 0.025).

In patients without known high cardioembolic risk (*n* = 348), SCCI was strongly associated with subsequent cardioembolism diagnosis (OR 3.00 [CI 1.58–5.73, *p* < 0.001]). No such association was present in non-SCCI.

**Conclusions:**

Acute or chronic SCCI are strongly associated with a cardioembolic origin.

## Background

Acute symptomatic cerebellar infarctions (CI) represent 2–3% of ischemic strokes [1, 2]. Most common stroke mechanisms are atherothrombosis (in situ occlusion of penetrating arteries or vertebral/basilar arteries), thromboembolism (artery-to-artery embolism), and embolism of cardiac origin.

In early reports studying patients with acute symptomatic CI, CI were divided into territorial (posterior inferior, anterior inferior, or superior cerebellar artery territories) and non-territorial CI (classically considered as borderzone CI, with an oblique orientation and a diameter of < 2 cm), with non-territorial infarctions having the same high rate of embolic mechanism as territorial infarctions [3–8]. The main causes of small CI were large-artery occlusive disease involving the vertebral/basilar arteries and embolism from cardiac source.

There are several concerns regarding the classification into large territorial vs. small non-territorial CI. For instance, some small infarctions may simply be small territorial infarctions (thus not necessarily occurring in a territorial border zone distribution) or internal cerebellar artery watershed infarctions. In addition, arterial perfusion territories vary considerably among individuals and vascular territories do not respect anatomical boundaries as is the case with fissures [9]. Small cortical cerebellar infarctions (SCCI) typically appear in an oblique fashion because they are small and because they are oriented orthogonally to the cerebellar fissures, whereas larger cortical cerebellar infarcts lose their oblique orientation with increasing size. The rare infarctions situated in the deep cerebellum typically lack oblique orientation [10, 11].

In a study analyzing 100 acute ischemic stroke (in any region) patients associated with chronic small CI, the presence of small CI (most frequently involving the cerebellar cortex) was mainly associated with embolism of cardiac source [10].

Recently, very small (< 1.5–2 cm, most often asymptomatic, chronic) CI have been found to be far more common than larger (often symptomatic) infarctions in a cohort study of patients with a history of arterial disease [11, 12]. Since these small infarctions typically occur in a distal (cortical) arterial territory, they are sometimes referred to as end-territorial infarctions whilst the smallest of them have recently become known as cortical cerebellar microinfarctions (i.e. cerebellar cortical infarct cavities corresponding to the remnants of small arterial cerebellar cortex infarction) [13]. In these studies, very small cortical cerebellar infarctions were associated with markers of atherothrombotic cerebrovascular disease [11, 14]. Another study showed cortical cerebellar microinfarctions in one third of patients with recent symptomatic vertebral artery stenosis [12]. These data suggest that these cortical cerebellar cavities are related to artery-to-artery embolism [11–16].

In reports studying lesion topography of acute infarctions in different TOAST stroke subtypes and in patients with atrial fibrillation (AF) detected by insertable cardiac monitors, cerebellar infarctions were not specifically analyzed [17, 18]. In another study assessing imaging characteristics of infarctions related to patent foramen ovale and AF, single acute infratentorial infarction was frequently observed in AF-related strokes, with the so-called cortico-subcortical lesion being the most frequently observed infratentorial lesion type [19]. However, in the latter study no analyses were performed in non-cardioembolic stroke patients.

Mainly, SCCI have been correlated with markers of artery-to-artery embolism. The goal of the present study was to correlate SCCI with cardiac embolism as well, by studying patients with acute symptomatic (anterior or posterior circulation) brain infarction showing associated acute or chronic SCCI on MRI.

## Methods

### Patients

We retrospectively analyzed consecutive adult patients, recruited between January 2016 and December 2016 in our center (Nîmes University Hospital, France), with acute symptomatic brain infarction confirmed by diffusion-weighted MRI. A total of 514 stroke patient episodes were screened. For 17 patients with more than one infarction episode during the recruitment period (16 patients with two episodes and one patient with three episodes), only the most recent episode was analyzed. Forty-five patients were excluded because only CT brain imaging was performed, and five patients because movement artifacts on MRI did not permit reliable analysis. In order to accurately analyze stroke mechanism, at least one complete intra- and extracranial vessel imaging modality and standard ECG was required for inclusion. Two patients were excluded because of missing extracranial vessel imaging, leaving a total of 452 patients for analysis.

Analyzed baseline characteristics were: sex, age, common cardiovascular risk factors (arterial hypertension, past or ongoing smoking, diabetes, hypercholesterolemia), history of symptomatic stroke, and history of AF.

Ethics approval was obtained according to local regulations. Informed consent requirements were waived.

### Diagnostic work-up

The TOAST classification was used for determining the stroke subtype. Recently, the clinical concept of embolic stroke of undetermined source (ESUS) was introduced to identify patients with non-lacunar cryptogenic ischemic strokes in whom embolism was the likely stroke mechanism [20]. Therefore, for some of the analyses, we also used the combined cardioembolic (CE)/undetermined etiology (UDE) as stroke subtype. In case of the presence of more than one stroke subtype (e.g. CE and LAA), the final stroke subtype was classified as UDE.

Craniocervical arteries were analyzed by duplex ultrasound, CT angiography (CTA), and/or gadolinium-enhanced MR angiography (MRA) according to the discretion of the treating physician. Based on analysis of vessel imaging, the presence of a vertebrobasilar > 50% stenosis or occlusion was noted (assessed by an experienced rater [ATS] blinded to parenchymal MRI and clinical data). When more than one vessel imaging modality was available, assessment was based on CTA (considered as most reliable vessel imaging) when performed. When CTA was unavailable and both MRA and duplex ultrasound were performed, vessel analysis was based on the imaging modality offering the most detailed information. Non-stenotic (< 50%) atherosclerotic plaques were not assessed.

Patients had ≥24 h ECG monitoring (acute stroke unit monitoring and/or Holter monitoring) unless refused by the patient. Echocardiography was proposed to all patients and performed in general two to three weeks after admission (i.e. during hospitalization when still hospitalized or in our outpatient clinic when discharged from the hospital in case of transient symptoms or minor stroke). In case of indication for urgent echocardiography (e.g. suspected intracardiac thrombus or infective endocarditis), echocardiography was performed during the hours or days following admission. Vessel imaging was performed during hospitalization in all patients.

### MRI

All patients underwent MRI, using a 1.5 T magnet (Ingenia, Philips, The Netherlands) using identical TE and TR parameters for each of the performed sequences. In case of technical problems with the 1.5 T MR scanner, a 3 T magnet (Skyra, Siemens, Erlangen, Germany) was used. A 1.5 T magnet MRI was used in 97% of patients and a 3 T magnet in the remainder. The acute stroke MRI protocol included diffusion-weighted imaging (b-values = 0 and 1000 s/mm^2^, TR 4280 ms, TE 97 ms), FLAIR (TR 10,000 ms, TE 140 ms), T2*-weighted (TR 950 ms, TE 23 ms), and intracranial 3-dimensional arterial TOF imaging (TR 25 ms, TE 7 ms). Due to time constraints in acute stroke care management, T1- and T2-weighted images were not performed as standard sequences in our center in patients eligible for intravenous thrombolysis or mechanical thrombectomy. Intra- and extracranial 3-dimensional gadolinium-enhanced MRA (TR 5 ms, TE 1.6 ms) were performed during the initial MRI when required for decision-making in the acute stroke phase.

### MRI analysis of acute symptomatic infarction and SCCI

Parenchymal MRI was analyzed by an experienced rater (ATS) blinded to clinical data and vessel imaging (i.e. the rater was also blinded to TOF-MRA and gadolinium-enhanced MRA sequences).

For the acute symptomatic infarction based on axial DWI, we assessed the infarction location (anterior, posterior, or mixed anterior-posterior circulation) and the presence of multiterritorial infarction (mixed anterior-posterior circulation or bilateral anterior circulation).

**Fig. 1:**
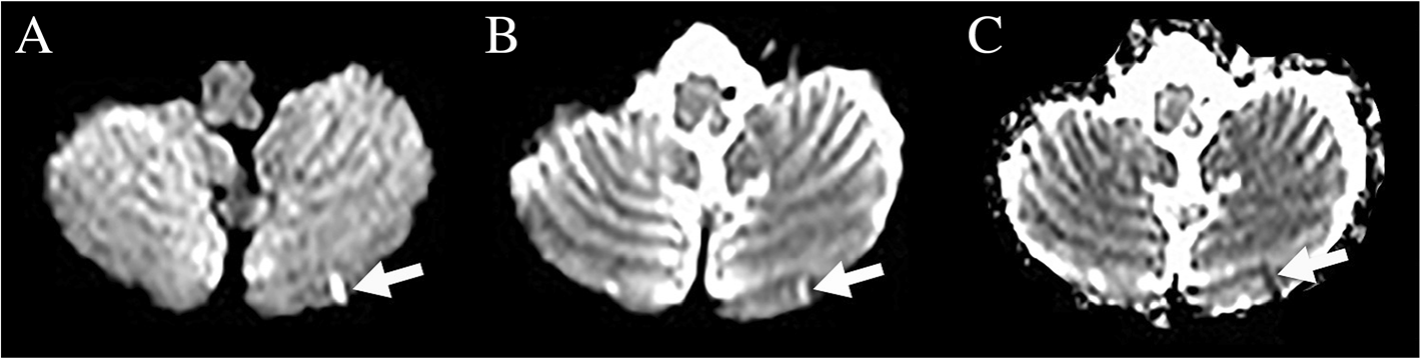
MRI showing a patient with acute SCCI, seen as hyperintensity on DWI B1000 (**a**) and B0 images (**b**) and as hypointensity on ADC map (**c**)

**Fig. 2:**
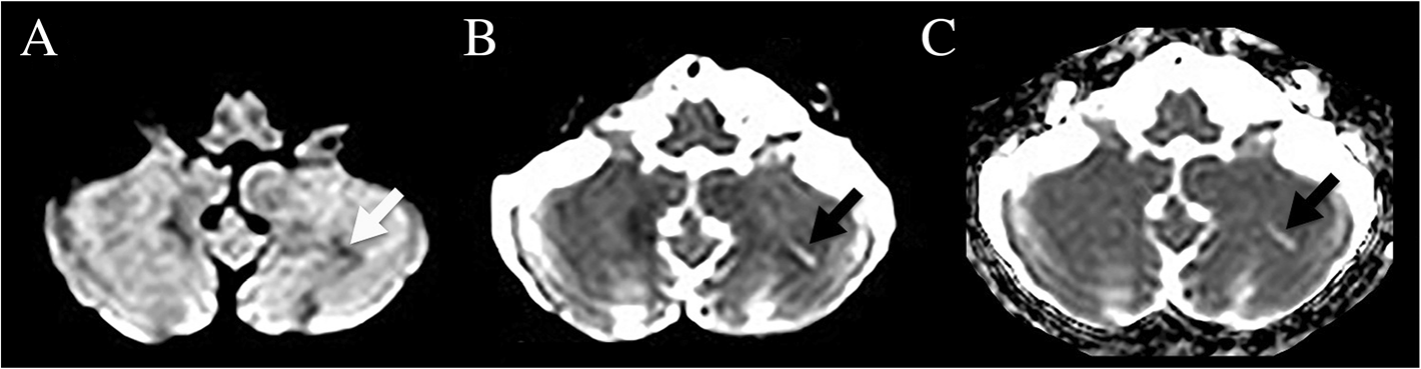
MRI showing a patient with chronic SCCI, seen as hypointensity on DWI B1000 (**a**) and as hyperintensity on B0 images (**b**) and ADC map (**c**)

To identify acute and chronic SCCI, we used axial DWI (B1000), B0, and ADC map images. SCCI was defined as a small (< 2 cm long) obliquely oriented, streak-like, longitudinal (the length more than twice the width of the lesion) CI. SCCI was considered to be acute when hyperintense on DWI and hypointense on ADC map (Fig. 1), and chronic when hyperintense on ADC map and B0 images and iso/hypointense on DWI (Fig. 2). In order to analyze whether CI with this specific size and form (SCCI) differed from other CI, we also assessed the presence of acute or chronic CI not corresponding to SCCI criteria (i.e. larger and/or without the specific orientation and form of SCCI, subsequently called non-SCCI). To study the consistency of the rater (ATS), assessment of 80 MRI scans was repeated by another rater (DR), showing good inter-rater agreement with a kappa value of 0.7 for the presence of SCCI. The exact location and the involved cerebellar artery territory of CI were not assessed.

### Statistical analyses

First we compared patients showing CI presence vs. absence, SCCI presence (with or without associated non-SCCI) vs. absence, and non-SCCI presence (with or without associated SCCI) vs. absence, and finally we directly compared patients with SCCI (with or without associated non-SCCI) vs. patients with non-SCCI (with or without associated SCCI), and patients with isolated SCCI vs. isolated non-SCCI. A pre-specified analysis was also performed in patients without known high risk of CE by excluding patients with AF history and acute multiterritorial infarction.

Fisher’s exact test and odd’s ratio (OR) were used to compare categorical variables, and Student *t* test was used to compare continuous variables between groups.

A multivariate logistic regression model was adjusted to fit SCCI and non-SCCI presence on dependent variables that were significant in a univariate way at a threshold of 0.2 and that were non-collinear between them.

## Results

A total of 452 patients were analyzed. General patient characteristics, diagnostic work-up data, and location and TOAST classification of acute symptomatic infarction are shown in Table 1.

**Table 1:** General characteristics, diagnostic work-up, lesion location and TOAST classification of acute symptomatic infarction (*n* = 452)

*General characteristics*	
Median age	72 (IQR 61–81)
Sex	61% men (*n* = 276)
Arterial hypertension	55% (*n* = 249)
Smoking	36% (*n* = 163)
Diabetes	23% (*n* = 104)
Hypercholesterolemia	38% (*n* = 172)
Brain infarction history	18% (*n* = 81)
AF history	12% (*n* = 54)
*Diagnostic work-up*	
*Cardiac*	
Standard ECG	100% (*n* = 452)
Prolonged (≥24 h) ECG	91% (*n* = 411)
-In hospital monitoring only	20% (*n* = 90)
-Holter monitoring only	16% (*n* = 72)
-Both in-hospital and Holter monitoring	55% (*n* = 249)
Echocardiography	71% (*n* = 321)
*Vessel imaging*	
-Duplex or CTA or gadolinium-enhanced MRA	100% (*n* = 452)
-Duplex only	8% (*n* = 36)
-CTA only	10% (*n* = 45)
-Gadolinium-enhanced MRA only	26% (*n* = 118)
Two vessel imaging modalities	42% (*n* = 190)
Three vessel imaging modalities	14% (*n* = 63)
*Location acute symptomatic infarction*	
-anterior circulation	66% (*n* = 298)
-posterior circulation	22% (*n* = 99)
-mixed anterior-posterior circulation	12% (*n* = 54)
-multiterritorial	15% (*n* = 68)
*TOAST classification*	
Cardioembolism	26% (*n* = 119)
-AF	83% (*n* = 98)
-known AF	45% (*n* = 53)
-newly discovered	38% (*n* = 45)
-other than AF cardioembolic cause	17% (*n* = 20)
Large-artery atherosclerosis	17% (*n* = 76)
Small vessel disease	10% (*n* = 46)
Stroke of undetermined etiology	41% (*n* = 187)
Stroke of other determined etiology	5% (*n* = 24)

Acute or chronic CI was present in 154 (34%), isolated SCCI in 55 (12%), isolated non-SCCI in 50 (11%), and mixed SCCI and non-SSCI in 49 patients (11%). SCCI and non-SCCI was acute in 3 and 8%, chronic in 19 and 11%, and mixed acute-chronic in 1 and 3% of the patients, respectively. In SCCI patients, mean SCCI number was 1.5 (range 1–5). Overall, vertebrobasilar stenosis > 50% was observed in 68 patients (15%). Mean duration of ECG monitoring was 2.7 days (range 1 to 23 days).

### Factors associated with presence of CI, SCCI, and non-SCCI

Association of CI, SCCI, and non-SCCI presence with baseline characteristics, vertebrobasilar stenosis/occlusion, infarction location, and stroke subtypes are shown in Table 2. CI, SCCI, and non-SCCI presence were each associated with AF history, acute posterior infarction, acute multiterritorial infarction, high proportion of CE and large-artery atherosclerosis stroke, and CE more frequent than other stroke subtypes. Only CI and SCCI were associated with older age, non-SCCI with infarction history, and CI and non-SCCI with vertebrobasilar stenosis/occlusion.

**Table 2:** Association of CI, SCCI, non-SCCI presence with baseline characteristics, vertebrobasilar stenosis/occlusion, infarction location, and stroke subtypes

	CI presence (*n* = 154) vs. CI absence (*n* = 298)		SCCI presence (*n* = 104) vs. SCCI absence (*n* = 348)		Non-SCCI presence (*n* = 99) vs. Non-SCCI absence (*n* = 353)	
Age (median)	75.5 vs. 70	*p* < 0.001*	78 vs. 71	*p* < 0.001*	74 vs. 71	*p* = 0.20
AF history	18% (*n* = 28) vs. 8% (*n* = 24)	*p* = 0.005*	18% (*n* = 19) vs. 9% (*n* = 31)	*p* = 0.021*	20% (*n* = 20) vs. 9% (*n* = 32)	*p* = 0.004*
Infarction history	23% (*n* = 35) vs. 15% (*n* = 45)	*p* = 0.051	24% (*n* = 25) vs. 19% (*n* = 66)	*p* = 0.056	23% (*n* = 23) vs. 16% (*n* = 56)	*p* = 0.036*
Vertebrobasilar stenosis/occlusion	22% (*n* = 34) vs. 11% (*n* = 33)	*p* = 0.003*	20% (*n* = 21) vs. 13% (*n* = 45)	*p* = 0.086	25% (*n* = 25) vs. 12% (*n* = 42)	*p* = 0.002*
Additional acute posterior infarction	57% (*n* = 88) vs. 22% (*n* = 66)	*p* < 0.001*	51% (*n* = 53) vs. 29% (*n* = 101)	*p* < 0.001*	64% (*n* = 63) vs. 26% (*n* = 92)	*p* < 0.001*
Multiterritorial infarction	25% (*n* = 39) vs. 10% (*n* = 30)	*p* < 0.001*	25% (*n* = 26) vs. 12% (*n* = 42)	*p* = 0.003*	26% (*n* = 26) vs. 12% (*n* = 42)	*p* < 0.001*
CE	39% (*n* = 60) vs. 20% (*n* = 60)	*p* < 0.001*	44% (*n* = 46) vs. 21% (*n* = 73)	*p* < 0.001*	42% (*n* = 42) vs. 22% (*n* = 78)	*p* < 0.001*
Newly discovered CE cause	17% (*n* = 26) vs. 11% (*n* = 33)	*p* = 0.10	25% (*n* = 26) vs. 11% (*n* = 40)	*p* = 0.001*	20% (*n* = 20) vs. 13% (*n* = 46)	*p* = 0.078
LAA	8% (*n* = 12) vs. 21% (*n* = 63)	*p* < 0.001*	6% (*n* = 6) vs. 20% (*n* = 70)	*p* < 0.001*	8% (*n* = 8) vs. 19% (*n* = 67)	*p* = 0.009*
OD	8% (*n* = 12) vs. 4% (*n* = 12)	*p* = 0.12	6% (*n* = 6) vs. 5% (*n* = 17)	*p* = 0.81	11% (*n* = 11) vs. 4% (*n* = 14)	*p* = 0.009*
SVD	8% (n = 12) vs. 11% (n = 33)	*p* = 0.25	6% (*n* = 6) vs. 12% (*n* = 42)	*p* = 0.10	10% (*n* = 10) vs. 10% (*n* = 35)	*p* = 1
UDE	37% (*n* = 57) vs. 44% (*n* = 131)	*p* = 0.19	38% (*n* = 40) vs. 42% (*n* = 146)	*p* = 0.50	29% (*n* = 29) vs. 45% (*n* = 159)	*p* = 0.006*
CE > LAA		*p* < 0.001*		*p* < 0.001*		*p* < 0.001*
CE > SVD		*p* = 0.005*		*p* = 0.001*		*p* < 0.001*
CE > UDE		*p* < 0.001*		*p* = 0.002*		*p* < 0.001*
UDE > LAA		*p* = 0.014*		*p* = 0.011*		*p* = 0.33
OD > UDE		*p* = 0.066		*p* = 0.79		*p* = 0.001*

*CI* cerebellar infarction, *SCCI* small cortical cerebellar infarction, *AF* atrial fibrillation, *CE* cardioembolic stroke subtype, *LAA* large large-artery atherosclerosis stroke subtype, *OD* other determined stroke subtype, *SVD* small vessel disease stroke subtype, *UDE* stroke of undetermined etiology; * is marked when *p* < 0.05

The first seven variables (from age to CE) represent the set of candidate variables for the multivariate model, whereas the other variables (below CE) were used for complementary analyses

Since a relatively large portion of patients (29%) lacked echocardiography and a minority of patients (9%) lacked ≥24 h ECG monitoring, we also performed a subgroup analysis of patients (*n* = 342) with both ≥24 h ECG monitoring and echocardiography, showing similar results (data not shown).

We also used a multivariate logistic regression model including stroke history, vertebrobasilar stenosis/occlusion, and CE stroke subtype as variables. The variables age, AF, acute posterior infarction, and acute multiterritorial infarction could not be included in the model because collinear to the variable of interest CE stroke (since all these parameters were significantly related [*p* < 0.05, Chi-2 test] to CE stroke subtype). SCCI presence was associated only with CE stroke subtype (*p* < 0.001, OR 2.85, 95% CI 1.78–4.56) (and not with stroke history, *p* = 0.16, OR 1.48, 95% CI 0.84–2.56; or vertebrobasilar stenosis/occlusion, *p* = 0.20, OR 1.47, 95% CI 0.8–2.64) whereas non-SCCI presence was associated with both CE stroke subtype (*p* < 0.001, OR 2.32, 95% CI 1.43–3.76) and vertebrobasilar stenosis/occlusion (*p* = 0.006, OR 2.28, 95% CI 1.28–4.02) (and not with stroke history, *p* = 0.10, OR 1.59, 95% CI 0.9–2.76).

### Comparison of patients with SCCI versus non-SCCI presence

In patients with CI, patients with SCCI presence were older (*p* = 0.046) than patients with non-SCCI presence, had less frequent history of symptomatic stroke (*p* < 0.001), and more frequent CE infarction (53% vs. 30%, *p* = 0.025). Patients with isolated SCCI (i.e. without associated non-SCCI) had more frequent CE or UDE stroke than patients with isolated non-SCCI (87% vs. 64% for combined CE/UDE stroke, *p* = 0.006). Sex, cardiovascular risk factors, AF history, vertebrobasilar stenosis/occlusion, infarction location did not differ between SCCI and non-SCCI groups. SCCI and non-SCCI groups had similar proportions of echocardiography and ≥ 24 h ECG monitoring performed.

### Analyses in patients without known high risk for CE stroke origin

In patients without known high CE risk (i.e. excluding patients with known AF or multiterritorial acute infarction, *n* = 348), after cardiac work-up for acute symptomatic infarction, CI and non-SCCI were not associated with subsequent CE diagnosis (*p* = 0.1 and *p* = 0.078, respectively) whereas SCCI was strongly associated (*p* = 0.001) with subsequent CE diagnosis (OR 3.00 [CI 1.58–5.73, *p* < 0.001]). SCCI presence showed an OR of 2.47 (CI 1.35–4.54, *p* = 0.0035) for combined CE/UDE stroke. No such association was present in patients with non-SCCI (OR 1.6 [CI 0.84–3.38, *p* = 0.15] for CE and OR 1.02 [CI 0.58–1.79, *p* = 0.95] for CE/UDE stroke).

## Discussion

Both SCCI and non-SCCI presence were associated with cardioembolism (i.e. AF history, acute multiterritorial stroke, and CE more frequent than large-artery atherosclerosis origin for the acute stroke). However, only non-SCCI presence was associated with vertebrobasilar stenosis/occlusion. When directly comparing SCCI and non-SCCI patients, only SCCI presence was associated with CE origin of stroke. These data are in favor of more frequent CE origin of stroke in patients with SCCI, and mixed CE/large-artery atherosclerosis origin in patients with non-SCCI. The highly significant and strong SCCI-CE stroke subtype association was most evident in stroke patients without probable CE (i.e. without known AF history or acute multiterritorial infarction) stroke origin, contrasting with non-SCCI where no such association was observed. SCCI (most frequently chronic on MRI) were seen in older patients and more often in patients without history of symptomatic infarction compared with non-SCCI. This was probably due to the fact that SCCI were defined as small CI lesions presenting with less severe or absence of neurological signs than larger non-SCCI lesions.

In our analyses regarding TOAST subtypes in the SCCI patients, results for UDE stroke origin were quite similar (although with a weaker association observed than for CE) to those of CE stroke subtype, suggesting potential cardioembolic stroke of undetermined source in these patients.

A major limitation of our study was the use of diffusion-weighted imaging to detect small CI lesions. Due to relative low spatial resolution of DWI and B0 images and ADC map compared to T1- and T2-weighted imaging, SCCI lesions were underestimated (although still highly prevalent) in our study. The advantage, however, is that these CI lesions could be assessed on the same sequences (i.e. DWI and B0 sequences, and ADC map) needed for acute infarction imaging.

In addition, no distinction was made for lesion size (< 2 cm) between acute and chronic SCCI. Since acute infarctions tend to shrink over time and retract in the chronic phase, some chronic CI might have been classified as SCCI (< 2 cm) whereas the corresponding infarction in the acute phase might have been larger than 2 cm (and thus would have been classified as non-SCCI if the infarction was observed in the acute phase). This effect might have led to possible underestimation of SCCI in the acute phase and overestimation of SCCI presence in the chronic phase in our study.

The relatively low portion of patients (71%) who underwent echocardiography in our study was probably explained by the delay of echocardiography (two to three weeks after admission) in case of non-urgent echocardiography (which was often scheduled after hospitalization in the outpatient clinic in patients with transient symptoms or minor stroke and associated relative short hospitalization period, tending not to show up for outpatient clinic echocardiography). In contrast, the vast majority of patients had ≥24 h ECG monitoring in our study (and more than half of the patients had both in-hospital and Holter monitoring). Since similar results were observed in subgroup analysis of patients with complete cardiac work-up (including ≥24 h ECG monitoring and echocardiography), and SCCI and non-SCCI patients had comparable cardiac examinations, the extent of cardiac examinations in our study probably did not interfere significantly with the results.

In our study, the lack of statistical significance for the association between SCCI and vertebrobasilar stenosis/occlusion might be related to the lack of study power since only a minority of patients (15%) showed vertebrobasilar stenosis/occlusion and to the fact that artery-to-artery embolism was probably underestimated in our study due to the criterium of 50% stenosis (excluding patients with small non-stenosing atherosclerotic plaques causing rupture, embolism, and SCCI) and that a minority (8%) of patients only had duplex ultrasound (permitting only partial evaluation of cervical vertebral artery segments).

In earlier reports analyzing small CI and associated stroke subtypes, both CE and large-artery atherosclerosis stroke subtypes were identified as frequent causes. In these series, only acute symptomatic CI patients were included [3–8]. Our study, including higher patient numbers and with most CI patients showing chronic and asymptomatic CI, showed preferential CE stroke subtype, especially in patients with SCCI. These SCCI may represent (often chronic and asymptomatic) intra- or inter-territorial end zone infarctions of CE origin.

In recent studies analyzing small, asymptomatic, chronic CI (resembling what we called SCCI in our study), association with markers of atherothrombotic cerebrovascular disease was observed suggesting artery-to-artery embolic origin of these cerebellar cortical cavities [11–16]. However, in these studies no details were given on the proportion of patients having had ≥24 h ECG monitoring (to detect the most frequent cause of cardioembolism, i.e. AF). In cohort studies of patients presenting with all kinds of arterial disease history, it is likely that ≥24 h ECG monitoring was only performed in a minority of patients, probably underestimating AF prevalence in these studies.

## Conclusions

Both acute and chronic SCCI have to be searched on MRI in acute stroke patients since SCCI presence may identify patients at high risk of CE stroke who might benefit from extensive cardiac work-up including prolonged/repeated ECG monitoring or insertable cardiac monitors.

## Abbreviations

AF: Atrial fibrillation
CE: Cardioembolic
CI: Cerebellar infarction
CTA: Computed tomography angiography
ESUS: Embolic stroke of undetermined source
MRA: Magnetic resonance angiography
OR: Odd’s ratio
SCCI: Small cortical cerebellar infarction
UDE: Undetermined etiology
